# Phytochemical Composition and Protective Effect of *Vernonanthura polyanthes* Leaf against In Vivo Doxorubicin-Mediated Toxicity

**DOI:** 10.3390/molecules27082553

**Published:** 2022-04-15

**Authors:** Jamira Dias Rocha, Marilia Elias Gallon, Abel Vieira de Melo Bisneto, Vanessa Cristiane Santana Amaral, Luciane Madureira de Almeida, Leonardo Luiz Borges, Lee Chen-Chen, Leonardo Gobbo-Neto, Elisa Flávia Luiz Cardoso Bailão

**Affiliations:** 1 Laboratório de Biotecnologia, Campus Central, Universidade Estadual de Goiás, Anápolis 75132-903, GO, Brazil; jamiradias@gmail.com (J.D.R.); amaral.vcs@gmail.com (V.C.S.A.); luciane.almeida@ueg.br (L.M.d.A.); leonardo.borges@ueg.br (L.L.B.); 2 Núcleo de Pesquisa em Produtos Naturais e Sintéticos de Ribeirão Preto, Universidade de São Paulo, Av. do Café s/n, Ribeirão Preto 14040-903, SP, Brazil; mariliagallon@hotmail.com.br (M.E.G.); gobbo@fcfrp.usp.br (L.G.-N.); 3 Laboratório de Radiobiologia e Mutagênese, Departamento de Genética, Instituto de Ciências Biológicas I, Universidade Federal de Goiás, Goiânia 74045-155, GO, Brazil; abelmelo8@gmail.com (A.V.d.M.B.); chenleego@yahoo.com.br (L.C.-C.); 4 Escola de Ciências Médicas e da Vida, Pontifícia Universidade Católica de Goiás, Goiânia 74605-010, GO, Brazil

**Keywords:** Cerrado, comet assay, micronucleus test, murine model, natural products

## Abstract

*Vernonanthura polyanthes* (Spreng.) A.J. Vega & Dematt. (syn.: *Vernonia polyanthes* Less) is popularly known as “assa-peixe” and its leaves are used in folk medicine mainly to treat respiratory diseases. In this study, we evaluated the cytogenotoxic and anticytogenotoxic potential of the *V. polyanthes* leaf aqueous extract (*Vp*LAE) and its *n*-butanol fraction (*n-*BF) in the presence or absence of doxorubicin (DXR) (pre-, co-, and post-treatments) on a murine model for 24 h or 120 h. The micronucleus test (MN) and the comet assay were used to assess the cytogenotoxic and anticytogenotoxic potential of *Vp*LAE and *n-*BF (250, 500, and 1000 mg/kg) administered via gavage to Swiss Webster mice. The chemical profiles of *Vp*LAE and *n-*BF were assessed by liquid chromatography coupled to mass spectrometry, and their metabolites were putatively identified. Lastly, the possible biological activities related to the (anti) cytogenotoxicity of the compounds were predicted using the PASS online webserver. The in vivo results showed that different doses of *Vp*LAE and *n-*BF did not present cytotoxic activity; however, the MN test revealed a slight mutagenic activity for the 24 h treatments. Moderate genotoxic effects were demonstrated for all treatments in the comet assay. Regarding anticytotoxicity and antimutagenicity, *Vp*LAE and *n-*BF presented a high cytoprotective potential against DXR toxic effects. In the co-treatment, *Vp*LAE reduced the DXR genotoxicity by ~27%, and *n*-BF did not demonstrate antigenotoxic potential. In contrast, an antigenotoxic effect was observed for both *Vp*LAE and *n*-BF in the pre- and post-treatments, reducing DXR genotoxicity by ~41% and ~47%, respectively. Chemical analysis of *Vp*LAE and *n-*BF showed the presence of eight phenolic compounds, including seven chlorogenic acids and a flavonoid. The PASS online tool predicted antimutagenic, anticancer, antineoplastic, chemoprotective, antioxidant, and radical scavenging activities for all constituents identified in *Vp*LAE and *n*-BF. *V. polyanthes* leaves presented a protective effect against DXR cytogenotoxicity. In general, *Vp*LAE and *n*-BF showed a greater antigenotoxic potential in the pre- and post-treatments. The metabolites putatively identified in *Vp*LAE and *n*-BF exhibited antioxidant and chemoprotective potential according to computational prediction analysis. Altogether, our results highlight the potential application of *V. polyanthes* to protect against toxic manifestations induced by DXR.

## 1. Introduction

The search for plant metabolites is constantly increasing because they are more eco-friendly and usually less toxic to normal cells. According to the Food and Drug Administration agency, 40% of the approved molecules are natural compounds or inspired by them [1]. Moreover, many plant species are considered a good source of natural antioxidants and could be used as a functional food [2,3,4].

*Vernonanthura polyanthes* (Spreng.) A.J. Vega & Dematt. (syn.: *Vernonia polyanthes* Less), popularly known as “assa-peixe”, is an Asteraceae family species [5]. *V. polyanthes* leaves are used in folk medicine to treat respiratory disorders, pneumonia, bronchitis, coughs, flu, and colds, as well as kidney diseases, uterine infections, ulcers, hypertension, leishmaniasis, fever, hemorrhages, and gastric conditions [6,7,8,9,10,11,12]. Although there are other species popularly called “assa-peixe”, such as *Vernonia brasiliana*, *Vernonia cognata* Less., and *Vernonia ferruginea* Less., only *V. polyanthes* is regulated for phytotherapeutic use, registered in the Brazilian Pharmacopoeia of Herbal Medicines as an expectorant, in the form of tea obtained by infusion of the dry leaves [13]. The presence of flavonoids, saponins, tannins, coumarins, triterpenes, sesquiterpene lactones, chlorogenic acids, and phenolic acids was demonstrated in extracts from *V. polyanthes* leaves [14,15,16,17,18].

The *V. polyanthes* leaf aqueous extract (*Vp*LAE) did not demonstrate toxicity, genotoxicity, or antigenotoxicity on *Drosophila melanogaster*. However, this extract potentiated the genotoxicity of doxorubicin (DXR) [19]. In contrast, *Vp*LAE was cytotoxic to *Allium cepa* cells and *Artemia salina* [14]. Similarly, *V. polyanthes* leaf hydroalcoholic extract (2000 mg/kg) was cytogenotoxic when administrated to mice [20]. *Vp*LAE and its three fractions (aqueous; *n*-butanol, n-BF; and ethyl acetate) showed cytogenotoxicity against human lymphocytes in vitro using a cell viability test and CometChip assay. However, when co-treated with DXR, *V. polyanthes* decreased DXR genotoxicity by ~15% [21].

DXR is a chemotherapeutic drug widely used in anticancer therapy; however, its use is limited by the increased oxidative stress caused by the drug [22,23]. It is already known that the redox balance disorder caused by DXR activates mechanisms that lead to cell damage, causing an increase in the production of free radicals and a decrease in endogenous antioxidants, inducing toxicity in various organs and tissues [22,23,24,25].

In this sense, it is important to study the effects of *V. polyanthes* and its interaction with DXR on a murine model. Initially, a micronucleus test (MN) and comet assay were performed, associated (pre-, co-, and post-treatments) or not with DXR on Swiss Webster mice, aiming to evaluate the cytogenotoxic and anticytogenotoxic potentials of *Vp*LAE and *n-*BF. Then, the chemical profiles of both were determined, and the putatively identified metabolites were submitted to computational prediction analysis to predict their biological activities.

## 2. Methodology

### 2.1. Botanical Material

*V. polyanthes* leaves were collected at the Central Campus, in Anápolis, Goiás, Brazil (S 16°23′0.16″/W 48°56′37.8″, 1073 m) in November 2018. The botanical identification of this specimen was carried out by Dr. Aristônio Magalhães Teles from the Federal University of Goiás and an exsiccate (N° 10512) was deposited in the Herbarium of the State University of Goiás. 

### 2.2. Infusion Preparation and Fractionation

The botanical material was subjected to drying at room temperature and pulverized in a knife mill E-625 (Tecnal Ltda, Piracicaba, SP, Brazil). Powdered material was stored sheltered from light and moisture for subsequent use. Initially, the powder from *V. polyanthes* leaves was subjected to quality parameters. The aqueous extract was prepared, according to Brazilian Pharmacopoeia (1st edition), by infusion of *V. polyanthes* (syn.: *Vernonia polyanthes* Less) leaves in the proportion of 0.02 g of dried and powdered plant material for each mL of water [13]. Samples were frozen at −18 °C and lyophilized. The solvent partitioning was obtained by percolation as previously described [21], resulting in three fractions (aqueous; *n*-BF; and ethyl acetate). The *n*-BF was evaporated under reduced pressure, and the dry extract and fraction were lyophilized and stored at −20 °C for further biological assays. The *n*-BF was selected to continue the experiments because it presented the highest total phenolic, flavonoids, and tannins contents, and the highest antioxidant activity, according to previous work [21].

### 2.3. Animals

Male *Mus musculus* (Swiss Webster) outbred mice, weighing between 20 to 30 g and aged from 7 to 12 weeks, were obtained from the Central Animal Facility of the Federal University of Goiás. This study was approved by the Animal Research Ethics Committee of the Federal University of Goiás (CEUA/UFG), protocol number 069/18 (Supplementary Materials).

### 2.4. In Vivo Experimental Procedures

This study used 140 mice (28 groups with 5 animals). Before the experiments were carried out, the animals remained acclimated for 7 days in the Laboratory of Radiobiology and Mutagenesis of the Federal University of Goiás. On these days, the animals were kept in polypropylene cages (Length: 40 cm, Width: 30 cm, and Height: 16 cm) with 5 animals in each cage lined with shavings changed every two days, and fed commercial feed and filtered water, both offered ad libitum. The animals were kept at room temperature, humidity 50% ± 20%, and a 12 h light/dark-light cycle. Doses of 250, 500, and 1000 mg/kg of *Vp*LAE and *n-*BF were selected for the micronucleus test in mouse bone marrow based on previous work [20]. The animals in Group 1 (negative control) received mineral water orally in the same volume used to administer the aqueous extract of *V. polyanthes*. The animals in Group 2 (positive control) received DXR administered intraperitoneally (ip) at 50 mg/kg p.c. Animals from Groups 3 to 28 received different treatments with *Vp*LAE or *n-*BF (Table 1). After 24 or120 h of treatment, the animals were euthanized by cervical dislocation. Bone marrow cells were obtained from the femurs of mice using 1 mL of fetal bovine serum and centrifuged at 300× *g* for 5 min to make slides for the MN test and the comet assay.

### 2.5. Micronucleus Test (MN)

Mouse bone marrow cells were used to make cell smears on glass slides. After drying, the smears were fixed in absolute methanol (CH_4_O) for 5 min, then stained in buffered Giemsa dye (dibasic sodium phosphate and monobasic sodium phosphate, pH 6.8). For each animal, four slides were prepared. As recommended [26], 4000 polychromatic erythrocytes (PCEs) were analyzed for each animal to determine the frequency of micronucleated polychromatic erythrocytes (MNPCE). Cytotoxicity and anticytotoxicity were evaluated by the ratio of PCE and normochromatic erythrocytes (NCE). Whereas genotoxicity and antigenotoxicity were assessed by the frequency of MNPCE concerning the total number of cells analyzed. The analysis of the slides was performed under an optical light microscope (Olympus BH-2, Tokyo, Japan, objective 100×).

### 2.6. Comet Assay

Microscope slides were previously coated with 1.5% agarose of normal melting point (1.5%). Subsequently, a solution containing 10 µL of bone marrow cells previously diluted in fetal bovine serum, and 120 µL of low melting point agarose at 0.5% and 37 °C, was placed on the agarose-precoated slides. The slides were then kept in the refrigerator, inside a foil-lined slide holder, containing lysis solution (2.5 M NaCl, 100 mM EDTA, 10 mM TRIS, 10% DMSO, 1% Triton X-100, and 1000 mL H_2_O, pH 10 at 4 °C overnight). After this period, the slides were transferred to a horizontal electrophoresis vat, containing electrophoresis buffer (30 mM NaOH, 1 mM EDTA, pH 13) at 5 ± 2 °C, where they remained for 30 min for DNA unwinding. Then, they were subjected to 300 mA and 1 V/cm for 30 min. After electrophoresis, the slides were immersed in a neutralizing buffer solution (0.4 M, Tris-HCl, pH 7.5) for 5 min. The slides were stained with 20 µL of Nucleic Acid Dye (Diamond^TM^, 10%) and covered with a coverslip. The capture of comet images was performed in a fluorescence microscope (Axio Imager^®^ A2 and Zen 2.3 software Carl Zeiss AG, Germany, with 510–560 nm excitation filter and 590 nm barrier filter, in a 20× objective). The software TriTek CometScore^TM^ (version 1.5) was used to assess DNA damage. In this software, pixel intensity in nucleoid images provides values corresponding to the estimation of genomic damage, which is expressed as arbitrary units (AU). Nucleoids with fragmented heads (hedgehogs) were not included in the analyses. The parameter adopted for quantifying DNA damage was the percentage of DNA in the tail.

### 2.7. Statistical Analysis

For the parameters PCE/NCE and MNPCE, the mean ± standard deviations were calculated for each group. Data distribution was checked for normality using the Shapiro-Wilk test. The different groups were compared using the parametric (one-way ANOVA) or nonparametric Kruskal-Wallis test followed by the Dunn’s multiple comparison test. The comet assay results are presented as the mean ± standard deviations, and the analysis of variance (one-way ANOVA) was performed, followed by Tukey’s multiple comparison test. Analyzes were performed using the GraphPad Prism software version 8.0.1. *p* < 0.05 was considered significant.

### 2.8. Chemical Profiles

For the LC-MS analyses, extracts of *Vp*LAE and *n-*BF were prepared using 10 mg of the dry powder of each extract or fraction and 1 mL of a MeOH:H_2_O (7:3, *v*:*v*) solution with hydrocortisone (10 mg/mL) as an internal standard. Extraction was performed in an ultrasonic bath for 10 min at room temperature. The extracts were filtered through a 0.20 mm PTFE membrane before analysis. Chemical profiles were obtained on an Accela UHPLC instrument (Thermo Scientific™, Waltham, MA, USA) with a diode array ultraviolet light detector (UV-DAD) coupled to an ExactiveTM Plus mass spectrometer (Thermo Scientific™, Waltham, MA, USA) with electrospray ionization source and orbitrap analyzer. Chromatograms were acquired in positive and negative ionization modes using a C18 Kinetex column (1.7 µm, XB-C18, 150 mm × 2.1 mm, Phenomenex) and an elution gradient of water and acetonitrile both with 0.1% of formic acid. All other chromatographic and spectroscopic parameters followed the methodology employed by Gallon et al. (2018b). Metabolites detected in *Vp*LAE and *n-*BF were putatively identified by comparing the spectroscopic data of each chromatographic signal with the data available in the *in-house* database of secondary metabolites reported for Vernonieae species.

### 2.9. Prediction of Activity Spectra for Substances 

The bioactivity of the metabolites identified in the *Vp*LAE and *n-*BF were predicted using the PASS online webserver (http://www.pharmaexpert.ru/passonline/, accessed on 20 May 2020). Pa and Pi estimate the probability of the substance to be active or inactive, respectively, for each type of activity from the database [27,28]. Therefore, the results presenting Pa > 0.7 and Pi < 0.05 were selected for the analysis. 

## 3. Results

### 3.1. Cytogenotoxic Evaluation

**Figure 1:**
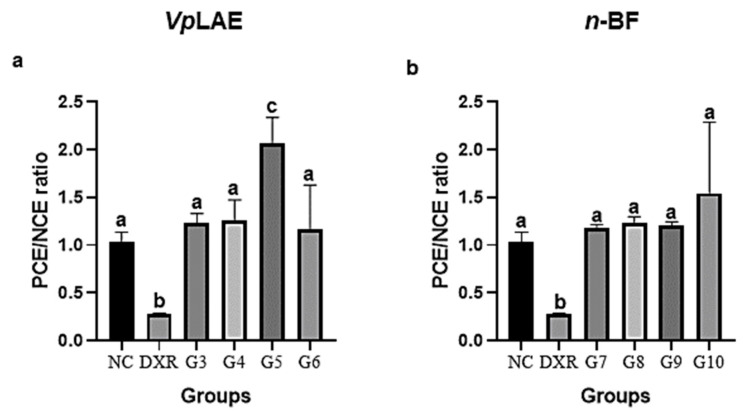
Evaluation of the cytotoxic potential of *Vernonanthura polyanthes* leaves aqueous extract (*Vp*LAE) and its *n-*butanol fraction (*n-*BF) on mouse bone marrow cells using the micronucleus test. The animals were treated with different concentrations of *Vp*LAE or its *n-*BF fraction. (**a**) Polychromatic erythrocytes (PCE)/normochromatic erythrocytes (NCE) ratio of animals treated with *Vp*LAE (G3: 250 mg/kg, G4: 500 mg/kg, G5: 1000 mg/kg for 24 h, and G6: 1000 mg/kg for 120 h). (**b**) PCE/NCE ratio of animals treated with *n-*BF (G7: 250 mg/kg, G8: 500 mg/kg, G9: 1000 mg/kg for 24 h, and G10: 1000 mg/kg for 120 h). NC: negative control (mineral water); DXR: positive control (doxorubicin: 50 mg/kg ip). Results are expressed as the mean ± standard deviation. The groups were compared by ANOVA (*Vp*lae) or Kruskal–Wallis (*n-*BF) followed by the respective Tukey or Dunn’s tests. Different letters indicate statistically significant differences between groups (*p* < 0.05).

**Figure 2:**
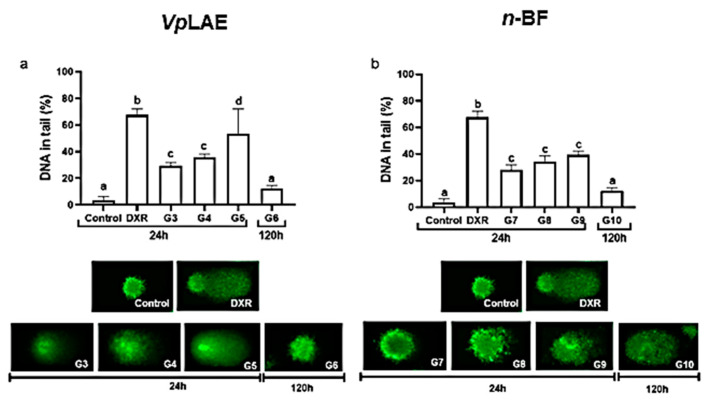
Evaluation of the genotoxic potential of *Vernonanthura polyanthes* leaves aqueous extract (*Vp*LAE) and its *n-*butanol fraction (*n-*BF) on mouse bone marrow cells using the comet assay. The animals were treated with different concentrations of *Vp*LAE or its *n-*BF fraction. The parameter used to assess genetic damage was % DNA in the tail. (**a**) *Vp*LAE (G3: 250 mg/kg, G4: 500 mg/kg, G5: 1000 mg/kg for 24 h, and G6: 1000 mg/kg for 120 h). (**b**) *n-*BF (G7: 250 mg/kg, G8: 500 mg/kg, G9: 1000 mg/kg for 24 h, and G10: 1000 mg/kg for 120 h). NC: negative control (mineral water); DXR: positive control (doxorubicin: 50 mg/kg ip). Results are expressed as the mean ± standard deviation. The groups were compared by ANOVA followed by the Tukey test. Different letters indicate statistically significant differences between groups (*p* < 0.05). Images below graphs are representative photomicrographs of nucleoids stained with Diamond^TM^ Nucleic Acid Dye. The images were captured using a fluorescence microscope (Axioplan-ImagingVR) and the Lucia software, with an excitation filter of 510–560 nm and a barrier filter of 590 nm (10× objective).

**Figure 3:**
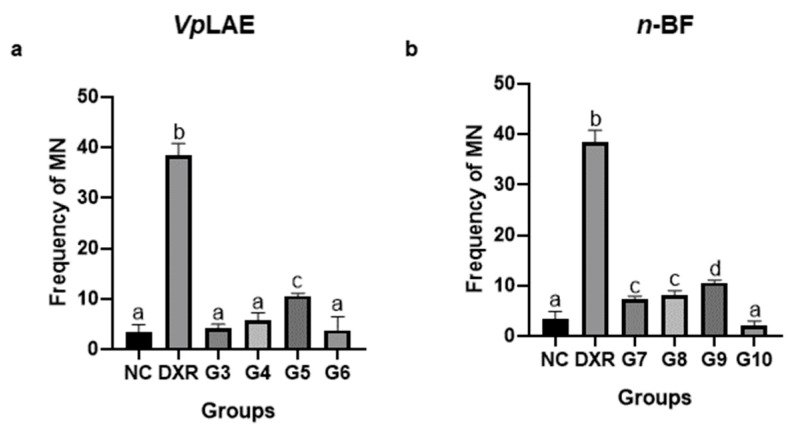
Evaluation of the mutagenic potential of *Vernonanthura polyanthes* leaves aqueous extract (*Vp*LAE) and its *n-*butanol fraction (*n-*BF) on mouse bone marrow cells using the micronucleus test. The animals were treated with different concentrations of *Vp*LAE or its *n-*BF fraction. (**a**) Micronucleated polychromatic erythrocyte frequency in animals treated with *Vp*LAE (G3: 250 mg/kg, G4: 500 mg/kg, G5: 1000 mg/kg for 24 h, and G6: 1000 mg/kg for 120 h). (**b**) Micronucleated polychromatic erythrocyte frequency in animals treated with *n-*BF (G7: 250 mg/kg, G8: 500 mg/kg, G9: 1000 mg/kg for 24 h, and G10: 1000 mg/kg for 120 h). NC: negative control (mineral water); DXR: positive control (doxorubicin: 50 mg/kg ip). Results are expressed as the mean ± standard deviation. The groups were compared by ANOVA followed by the Tukey test. Different letters indicate statistically significant differences between groups (*p* < 0.05).

**Figure 4:**
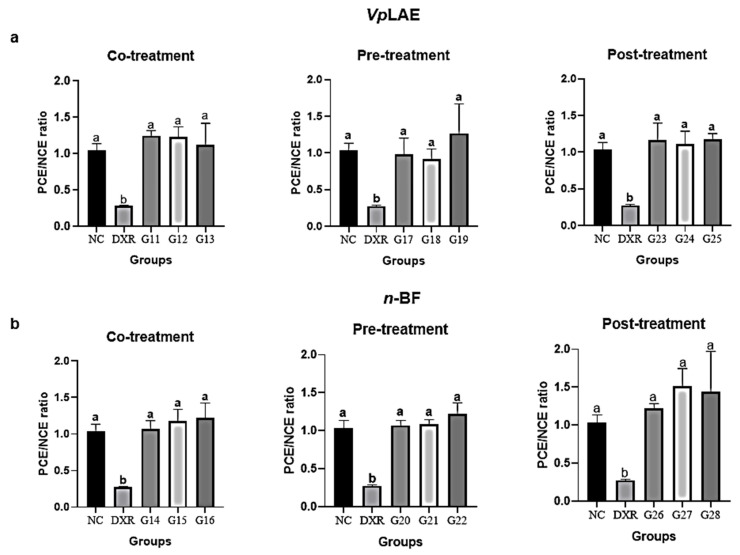
Evaluation of the anticytotoxic potential of *Vernonanthura polyanthes* leaves aqueous extract (*Vp*LAE) and its *n-*butanol fraction (*n-*BF) on mouse bone marrow cells using the micronucleus test. The animals were treated with different concentrations of *Vp*LAE or its *n-*BF fraction associated with the positive control doxorubicin (DXR; 50 mg/kg ip). (**a**) Polychromatic erythrocytes (PCE)/normochromatic erythrocytes (NCE) ratio of animals treated with *Vp*LAE (G3: 250 mg/kg, G4: 500 mg/kg, G5: 1000 mg/kg for 24 h, and G6: 1000 mg/kg for 120 h). (**b**) PCE/NCE ratio of animals treated with *n-*BF (G7: 250 mg/kg, G8: 500 mg/kg, G9: 1000 mg/kg for 24 h, and G10: 1000 mg/kg for 120 h). NC: negative control (mineral water). Results are expressed as the mean ± standard deviation. The groups were compared by ANOVA followed by the Tukey test. Different letters indicate statistically significant differences between groups (*p* < 0.05).

**Figure 5:**
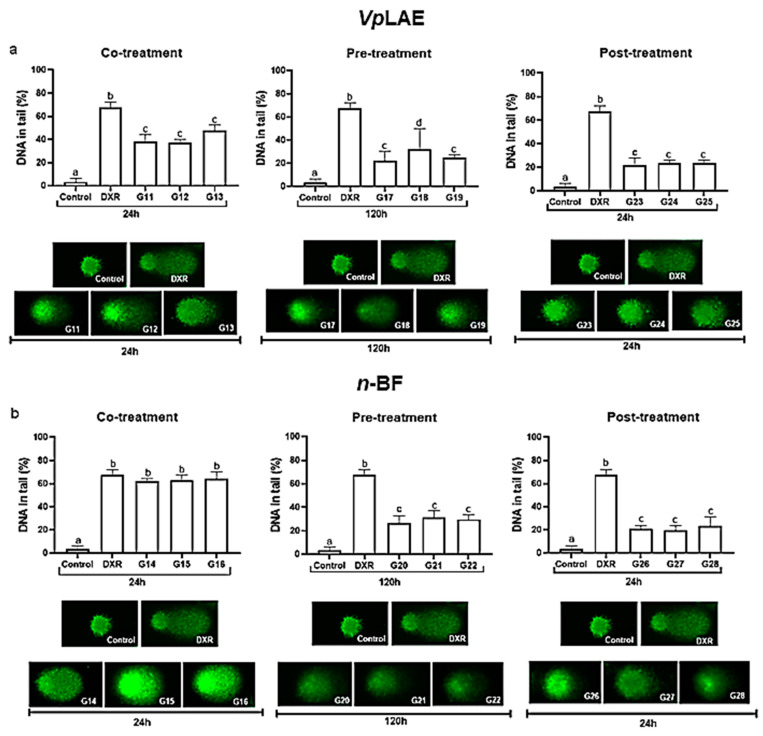
Evaluation of the antigenotoxic potential of *Vernonanthura polyanthes* leaves aqueous extract (*Vp*LAE) and its *n-*butanol fraction (*n-*BF) on mouse bone marrow cells using the comet assay. The animals were treated with different concentrations of *Vp*LAE or its *n-*BF fraction associated with the positive control doxorubicin (DXR; 50 mg/kg ip). The parameter used to assess genetic damage was % DNA in the tail. (**a**) *Vp*LAE (G3: 250 mg/kg, G4: 500 mg/kg, G5: 1000 mg/kg for 24 h, and G6: 1000 mg/kg for 120 h). (**b**) *n-*BF (G7: 250 mg/kg, G8: 500 mg/kg, G9: 1000 mg/kg for 24 h, and G10: 1000 mg/kg for 120 h). NC: negative control (mineral water). Results are expressed as the mean ± standard deviation. The groups were compared by ANOVA followed by the Tukey test. Different letters indicate statistically significant differences between groups (*p* < 0.05). Images below graphs are representative photomicrographs of nucleoids stained with Diamond^TM^ Nucleic Acid Dye. The images were captured using a fluorescence microscope (Axioplan-ImagingVR) and Lucia software, with an excitation filter of 510–560 nm and a barrier filter of 590 nm (10× objective).

**Figure 6:**
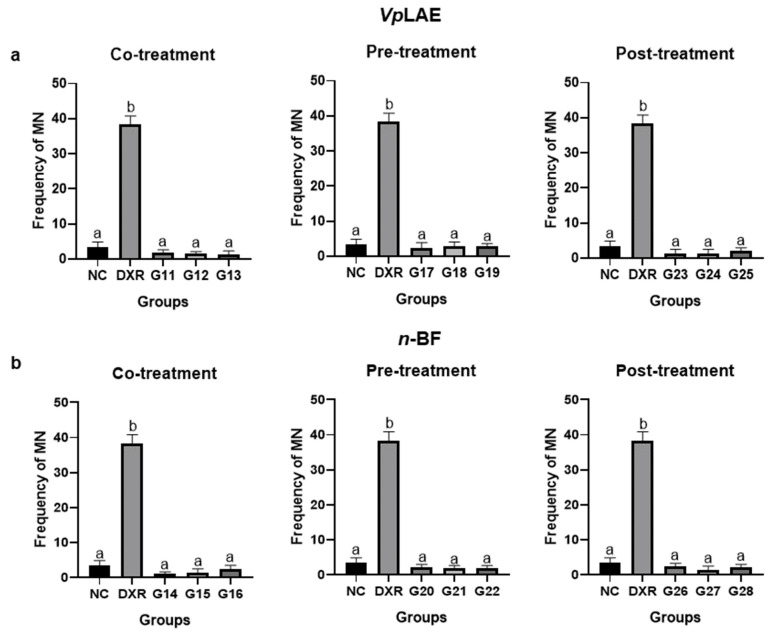
Evaluation of the antimutagenic potential of *Vernonanthura polyanthes* leaves aqueous extract (*Vp*LAE) and its *n-*butanol fraction (*n-*BF) on mouse bone marrow cells using the micronucleus test. The animals were treated with different concentrations of *Vp*LAE or its *n-*BF fraction associated with the positive control doxorubicin (DXR; 50 mg/kg ip). (**a**) Micronucleated polychromatic erythrocyte frequency in animals treated with *Vp*LAE (G3: 250 mg/kg, G4: 500 mg/kg, G5: 1000 mg/kg for 24 h, and G6: 1000 mg/kg for 120 h). (**b**) Micronucleated polychromatic erythrocyte frequency in of animals treated with *n-*BF (G7: 250 mg/kg, G8: 500 mg/kg, G9: 1000 mg/kg for 24 h, and G10: 1000 mg/kg for 120 h). NC: negative control (mineral water). Results are expressed as the mean ± standard deviation. The groups were compared by ANOVA followed by the Tukey test. Different letters indicate statistically significant differences between groups (*p* < 0.05).

*Vp*LAE and *n-*BF did not present cytotoxicity against mice bone marrow cells (Figure 1) and presented genotoxicity after acute (24 h) exposition to all concentrations tested in this work (250, 500, and 1000 mg/kg) (Figure 2). The genotoxicity observed at the highest concentration of *Vp*LAE (1000 mg/kg, G5) was similar to that observed for the positive control (DXR) (Figure 2a). Although *Vp*LAE and *n-*BF presented a genotoxic potential on mice bone marrow cells, these materials did not present or presented a mild mutagenicity potential (Figure 3). As expected, animals treated with DXR (positive control) showed cytogenotoxic effects compared to the negative control (Figure 1, Figure 2, Figure 3, Figure 4, Figure 5 and Figure 6).

### 3.2. Anticytogenotoxic Evaluation

The evaluation of *Vp*LAE and *n-*BF anti-cytogenotoxicity in the presence of the DXR positive control (co-, pre-, and post-treatments) was also performed. *Vp*LAE and *n-*BF were anti-cytotoxic in all doses (250, 500, and 1000 mg/kg) and in different treatment schemes used in this work (Figure 4). The PCE/NCE ratios in treatments of DXR + *Vp*LAE or *n-*BF were restored to the ratio obtained in the negative control (Figure 4). Regarding antigenotoxicity, *Vp*LAE reduced the DXR genotoxicity by ~27% during co-treatment (Figure 5a). In contrast, *n-*BF did not demonstrate anti-genotoxic potential in the co-treatment with DXR (Figure 5b). Regarding pre-treatment, *Vp*LAE and *n-*BF reduced the genotoxicity of DXR by ~41% (Figure 5). In the post-treatment, *Vp*LAE and *n-*BF also reduced the genotoxicity of DXR by ~47% (Figure 5). Moreover, the association of *Vp*LAE or *n-*BF with DXR in the co-, pre-, and post-treatments showed a significant reduction in the frequency of MN/PCE when compared to the positive control (Figure 6), revealing a relevant anti-genotoxic effect of *V. polyanthes*.

### 3.3. Chemical Profiles of VpLAE and n-BF

**Figure 7:**
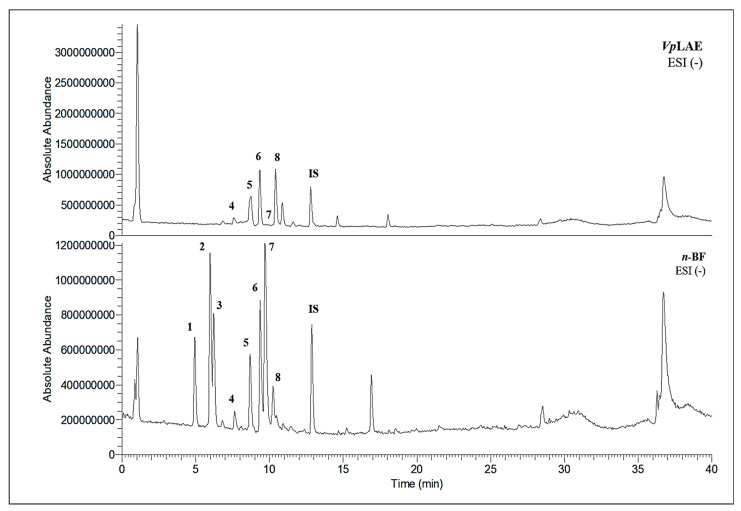
Total ion chromatograms (TIC) of *Vernonanthura polyanthes* leaf aqueous extract (*Vp*LAE) and its *n*-butanol fraction (*n*-BF) analyzed by UHPLC-UV-MS (Orbitrap) in the negative ionization mode. **1**, 3-*O*-caffeoylquinic acid; **2**, 5-*O*-caffeoylquinic acid; **3**, 4-*O*-caffeoylquinic acid; **4**, 5-*O*-feruloylquinic acid; **5**, rutin; **6**, 3,4-di-*O*-caffeoylquinic acid; **7**, 3,5-di-*O*-caffeoylquinic acid; **8**, 4,5-di-*O*-caffeoylquinic acid; **IS**, internal standard (hydrocortisone 10 mg/mL).

**Figure 8:**
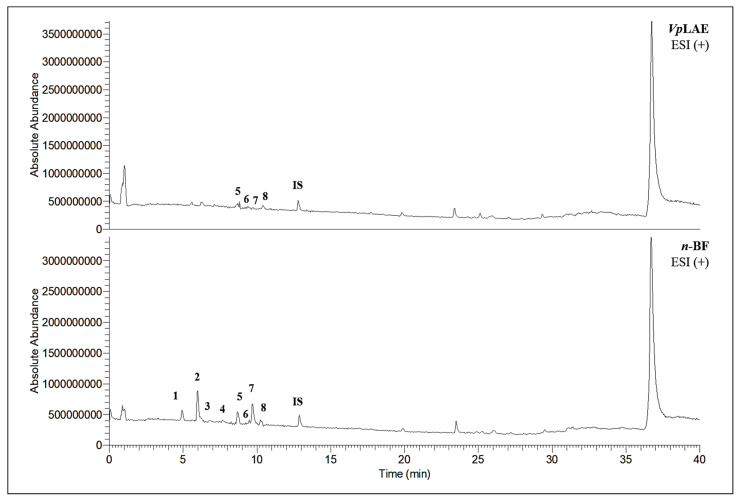
Total ion chromatograms (TIC) of *V. polyanthes* leaf aqueous extract (*Vp*LAE) and its *n*-butanol fraction (*n*-BF) analyzed by UHPLC-UV-MS (Orbitrap) in the positive ionization mode. **1**, 3-*O*-caffeoylquinic acid; **2**, 5-*O*-caffeoylquinic acid; **3**, 4-*O*-caffeoylquinic acid; **4**, 5-*O*-feruloylquinic acid; **5**, rutin; **6**, 3,4-di-*O*-caffeoylquinic acid; **7**, 3,5-di-*O*-caffeoylquinic acid; **8**, 4,5-di-*O*-caffeoylquinic acid; **IS**, internal standard (hydrocortisone 10 mg/mL).

LC-MS analyses of *Vp*LAE and *n*-BF confirmed the presence of phenolic compounds (flavonoids and chlorogenic acids) typically reported in *V. polyanthes* (Table 2 and Supplementary Table S1). The compounds 5-*O*-feruloylquinic acid, quercetin 3-*O*-rutinoside, 3,4-di-*O*-caffeoylquinic acid, 3,5-di-*O*-caffeoylquinic acid, and 4,5-di-*O*-caffeoylquinic acid were putatively identified in both the *Vp*LAE and *n-*BF samples. Whereas 3-*O*-caffeoylquinic acid, 5-*O*-caffeoylquinic acid, and 4-*O*-caffeoylquinic acid were detected in higher relative abundance in the *n-*BF. Accordingly, all the detected *O*-caffeoylquinic acids and di-*O*-caffeoylquinic acids exhibited greater relative abundances in *n-*BF (Figure 7 and Figure 8).

### 3.4. Biological Activity Prediction of Identified Secondary Metabolites

Potential antimutagenic, anticancer, antineoplastic, chemoprotective, antioxidant, and radical scavenging effects were attributed to all metabolites putatively identified in *Vp*LAE and *n-*BF by computational prediction analysis using the PASS online tool (Table 3). Moreover, the identified molecules are predicted modulators of enzymes involved in antioxidant processes such as HMOX1, lipid peroxidase inhibitors, aryl sulfotransferase, β-glucuronidase, α-glucosidase, UDP-glucuronosyl transferase, glutathione-disulfide reductase, and different P450 isoforms (CYP1A, CYP1A1, CYP3A4, CYP2C9, CYP3A) (Table 4). Among the metabolites detected in *Vp*LAE and *n-*BF, quercetin-3-*O*-rutinoside (rutin) seems to be the most active considering the analyzed antioxidant and chemopreventive parameters provided by the PASS online tool (Table 3 and Table 4).

## 4. Discussion

In this study, the MN test and the comet assay in mouse bone marrow were performed to assess the cytotoxic, genotoxic, and mutagenic potential of *Vp*LAE and *n-*BF. Moreover, the anticytogenotoxic potential of *Vp*LAE and *n-*BF was evaluated in the presence of DXR, a widely used chemotherapy agent. The micronucleus test in mammals assesses the cytotoxic and mutagenic effects of chemical or physical agents, providing a system to detect cytogenetic damage resulting from clastogenic or aneugenic activity [29,30]. The comet assay (single-cell gel electrophoresis) is a methodology used to measure deoxyribonucleic acid (DNA) strand breaks in cells [31,32,33,34]. Both approaches can determine the antigenotoxic potential of a plant extract or isolated compound [35,36,37]. 

The animals treated with *Vp*LAE and *n-*BF (250, 500, and 1000 mg/kg) did not promote a PCE/NCE ratio reduction compared to the negative control group, revealing the cytotoxic activity absence of *V. polyanthes* leaves. These results are consistent with our previous study using the wing recombination and somatic mutation test (SMART/wing), which revealed the absence of cytotoxic activity of the aqueous extract of *V. polyanthes* leaves (0.25–1 mg/mL) on *Drosophila melanogaster* [19]. In contrast, when the *V. polyanthes* leaf hydroalcoholic extract (1000, 1500, and 2000 mg/kg) was administrated to mice, the relationship between PCE and NCE was significantly reduced in all treatments, regardless the exposition time (24 and 48 h) or gender, indicating cytotoxicity of the *V. polyanthes* leaf hydroalcoholic extract [20]. *V. polyanthes* leaves were also cytotoxic to human lymphocytes, sarcoma-180 cells, *Allium cepa* cells, and *Artemia salina* [14,16,21]. The mode of action of plant extracts may vary depending on different model systems and chemical compositions [38,39,40]. So, these contrasting results could be explained by the model used to study cytotoxicity (if it is a metabolizing system or not), the type of solvent used to prepare the extracts, and the part of the plant used [38,40]. 

The comet assay has several applications in testing new chemicals, particularly genotoxicity or DNA repair damage evaluation [32,33]. *Vp*LAE and *n-*BF showed genotoxicity for all concentrations used (250, 500, and 1000 mg/kg) after 24 h exposition. Likewise, *Vp*LAE and its fractions (aqueous, *n-*butanol, and ethyl acetate) demonstrated genotoxic activity on human lymphocytes [21]. In contrast, when the hydroalcoholic extract of *V. polyanthes* leaves (1000, 1500, and 2000 mg/kg) was administrated to mice, just the highest dose tested was demonstrated to be genotoxic [20]. Similarly, the absence of genotoxic activity of *V. polyanthes* was verified on *D. melanogaster* and *A. cepa* at the studied conditions [14,19]. These discrepant results highlight the importance of using different models to study plant derivatives.

Slight mutagenicity was observed for some groups treated with *Vp*LAE (G5) and *n-*BF (G7–G9). On the other hand, the aqueous extract of *V. polyanthes* did not show mutagenicity at the concentrations used (0.25–1 mg/mL) on *D. melanogaster* using the wing somatic mutation and recombination test (SMART-wing) [19]. *V. polyanthes* aqueous extract did not also show a mutagenic effect on *A. cepa* meristematic cells [14]. The hydroalcoholic extract of *V. polyanthes* leaves (ethanol/water, 70/30 *v*/*v*) was mutagenic for mice only at the highest dose used (2000 mg/kg). The low and intermediate doses (1000 and 1500 mg/kg) were not mutagenic [20]. This discrepancy between the results may be associated with different models tested and the different solvents used to prepare the *V. polyanthes* extract (water or ethanol). The polarity-dependent increase in antioxidant activity may indicate that polar solvents are more likely to extract antioxidant compounds [41]. One of the most suitable solvents for aqueous mixtures is ethanol because it maximizes polyphenols extraction and is safe for human consumption [21,42].

This study also evaluated the anticytotoxic and antigenotoxic potential of *Vp*LAE and *n-*BF against DXR, a widely used chemotherapeutic, capable of performing single and double breaks in DNA. This drug is part of one of the most effective groups of antineoplastics used in current clinical practice. However, its use is limited by chronic and acute toxic side effects and susceptibility to numerous drug interactions [25,43,44,45]. The antitumor and toxic effects of DXR contribute to the production of free radicals and to the occurrence of oxidative stress, which can occur in four different ways: (i) semiquinone production; (ii) activation of NAD (P) H oxidases (NOXs); (iii) a non-enzymatic mechanism; and (iv) the generation of DXR metabolism products [24,46]. The generated oxidative stress confers non-specific cytotoxicity, leading to undesirable effects of chemotherapy with DXR [47]. DXR-mediated generation of excessive free radicals caused by increased free radical production and decreased endogenous antioxidants plays an important role in the pathogenesis of induced toxicity in various organs and tissues. DXR can also induce apoptosis and hyperlipidemia [24,25]. One of the oxidative stress reduction strategies has been the combination of the drug together with an antioxidant agent [25,44]. In this context, phytochemical compounds have been described as an alternative to mitigate the harmful effects caused by DXR since metabolites from natural products have shown to be promising in overcoming the limitations of DXR in pre-clinical models such as chemosensitizers, chemoresistance inhibitors, and protectors chemotherapeutics in different types of cancer [48]. 

The association of DXR with *Vp*LAE or *n-*BF in the co-, pre-, and post-treatments demonstrated an anticytotoxic potential of *V. polyanthes*. In contrast, the cytotoxicity of DXR was potentiated by *Vp*LAE and its aqueous, *n-*butanol, and ethyl acetate fractions (0.25–1 mg/mL) on human lymphocytes during co-treatment [21]. In vivo toxicity assessment involves important factors related to the dynamics of the accumulation of substances in cells that can occur through electron transport, absorption and diffusion, apoptosis process, production of reactive oxygen molecules, and biotransformation of molecules by intracellular/extracellular via activation of specific enzymes. The cell’s communication with its environment is a key factor in differentiating between in vivo and in vitro models [49].

The antigenotoxic potential of *Vp*LAE and *n-*BF (in the presence of DXR) was also evaluated in co-, pre-, and post-treatment schemes by comet assay. The results revealed that *Vp*LAE and *n-*BF reduced DXR genotoxicity by ~38%. The exception is *n-*BF co-treated with DXR, which could not protect the DNA. Similar results were observed for *Vp*LAE and its fractions (aqueous, ethyl acetate, and *n-*butanol) co-treated with DXR in human lymphocytes. *V. polyanthes* decreased DXR genotoxicity by ~15% [21]. However, previous results with *D. melanogaster* showed that *Vp*LAE potentiated DXR genotoxicity when both were administered in a co-treatment regimen [19].

The association of *Vp*LAE and *n-*BF with DXR in co-, pre-, and post-treatments showed a significant reduction in the frequency of MN in erythrocytes from the bone marrow of mice, showing a protective effect against damage caused by DXR. Unlike this result, tests conducted to evaluate the genotoxic effects using the wing recombination and somatic mutation test (SMART-wing) showed that the association of the aqueous extract of *V. polyanthes* with DXR potentiated its mutagenic effect, increasing the number of mutations in *D. melanogaster* somatic cells [19]. The observed differences may be associated with the model used to conduct the studies (murine x insect), highlighting the importance of using different study models during the pre-clinical phase of drug development.

This study also investigated secondary metabolites in *Vp*LAE and *n-*BF to infer their association with the anti-cytogenotoxic activity against the redox effect generated by DXR. Our LC-MS results of *Vp*LAE and *n-*BF confirmed the presence of phenolic compounds abundantly found in plants [50,51,52,53]. We putatively identified eight phenolic compounds, including seven phenols identified as chlorogenic acids and a flavonoid identified as quercetin 3-*O*-rutinoside (rutin). The presence of these metabolites was observed for other species of the genus *Vernonia* [54,55]. Flavonoids are known for their antioxidant potential aiding in the capture and scavenging of radicals, as well as being one of the most widely found secondary metabolites in medicinal plants [23,50,51,53]. In this sense, due to their radical scavenging properties, flavonoids may act in the redox effect mediated by DXR in the generation of cellular oxidative stress. The phenolic compounds noted here, more specifically the chlorogenic acids, have been widely studied in recent years regarding their effects and redox effects in various cell types [45]. 

Recently, the antioxidant potential of *V. polyanthes* leaves aqueous extract and its fractions (aqueous, *n-*butanol, and ethyl acetate) was investigated by the DPPH method showing great antioxidant potential for the extract and the *n-*butanol fraction [21]. In this sense, the metabolites detected in *Vp*LAE and *n-*BF may contribute to the prevention of DXR-induced overproduction of free radicals, protecting the bone marrow cells of mice. Phenolic compounds have hydroxyl groups, which can help in the process of scavenging free radicals by direct proton transfer. ROS elimination occurs in proportion to the number of functional hydroxyl groups present within each molecule [56,57]. There is growing evidence that compounds with antioxidant properties can remove ROS before these species react with DNA resulting in a mutation [56,58]. In this way, metabolites in plant extracts can increase the maintenance of DNA structure and modulation of DNA metabolism and repair, minimizing the redox effect of toxic compounds [59]. 

A high degree of correlation was found between the in vivo and in silico results. Among the metabolites detected in *Vp*LAE and *n-*BF, rutin stands out as a potential antioxidant and chemopreventive molecule against DXR-mediated cytogenotoxicity. The result of the PASS online prediction tool showed that rutin might be able to enhance *hmox1* expression. Hmox1 is an antioxidant enzyme produced to respond to oxidative stress [60,61]. Hmox1 activation inhibits lipid peroxidation, preventing cell damage caused by DXR [60,62]. It had been demonstrated that rutin attenuated the toxic effects caused by DXR in cardiac and renal tissues via improvement of the antioxidant state of cells [63]. Rutin administration also decreased DXR-induced heart failure, inhibiting excessive autophagy and apoptosis [64]. This suggests that using antioxidant agents, such as flavonoids, in combination with DXR may reduce or inhibit DXR-induced side effects. 

Moreover, rutin is a predicted CYP inducer. The cytochrome P450 enzymes metabolize xenobiotics, participating mainly in the conversion of toxic substances into more polar and water-soluble metabolites to be rapidly excreted, preventing cytotoxic and genotoxic effects [65]. However, during the biotransformation process, various unstable and reactive intermediates that react with DNA can be formed, causing genotoxicity and cell damage [65,66,67]. In this sense, it can be suggested that the moderate genotoxicity presented for the *Vp*LAE (G3, G4, G5, and G6) and the *n-*BF (G7, G8, G9, and G10), as well as the mild mutagenicity demonstrated for the G5 group (*Vp*LAE) and the groups G7, G8 and G9 (*n-*BF), could be a result of the CYP family induction. In addition, since the cytochrome P450 enzymes catalyze many metabolic reactions involving xenobiotics [66], these enzymes may also have acted in the potent antigenotoxic and antimutagenic activity observed for *Vp*LAE and *n-*BF.

## 5. Conclusions

*Vp*LAE and *n-*BF in co-, pre-, and post-treatments significantly inhibited DXR toxicity, protecting the mouse bone marrow cells against the cytotoxic, genotoxic, and mutagenic effects of DXR. This cytoprotective activity may be correlated with the antioxidant potential of phenolic compounds present in *Vp*LAE and *n-*BF. Furthermore, both *Vp*LAE and *n-*BF did not demonstrate to be cytotoxic at the concentrations used. Thus, studies about the association of DXR with natural antioxidants are encouraged to reduce the level of oxidative stress generated by the drug; that is, its side effects.

## Figures and Tables

**Table 1:** Experimental design of the micronucleus test and comet assay.

Animal Groups	Dose	Number of Animals (n)	Exposure Time
	**Controls**		
**G1**	NC (H_2_O)	5	24 h
**G2**	PC (DXR 50 mg/kg ip)	5	24 h
Genotoxicity			
	***Vp*** **LAE**		
**G3**	250 mg/kg	5	24 h
**G4**	500 mg/kg	5	24 h
**G5**	1000 mg/kg	5	24 h
**G6**	1000 mg/kg	5	120 h
	***n-*** **BF**		
**G7**	250 mg/kg	5	24 h
**G8**	500 mg/kg	5	24 h
**G9**	1000 mg/kg	5	24 h
**G10**	1000 mg/kg	5	120 h
Co-treatment			
	***Vp*** **LAE**		
**G11**	DXR + 250 mg/kg	5	24 h
**G12**	DXR + 500 mg/kg	5	24 h
**G13**	DXR +1000 mg/kg	5	24 h
	***n-*** **BF**		
**G14**	DXR + 250 mg/kg	5	24 h
**G15**	DXR + 500 mg/kg	5	24 h
**G16**	DXR + 1000 mg/kg	5	24 h
Pre-treatment			
	***Vp*** **LAE**		
**G17**	250 mg/kg + DXR	5	120 h
**G18**	500 mg/kg + DXR	5	120 h
**G19**	1000 mg/kg + DXR	5	120 h
	***n-*** **BF**		
**G20**	250 mg/kg + DXR	5	120 h
**G21**	500 mg/kg + DXR	5	120 h
**G22**	250 mg/kg + DXR	5	120 h
Post-treatment			
	***Vp*** **LAE**		
**G23**	DXR + 250 mg/kg	5	24 h
**G24**	DXR + 500 mg/kg	5	24 h
**G25**	DXR + 1000 mg/kg	5	24 h
	***n-*** **BF**		
**G26**	DXR + 250 mg/kg	5	24 h
**G27**	DXR + 500 mg/kg	5	24 h
**G28**	DXR + 1000 mg/kg	5	24 h

NC: Negative control; PC: positive control; DXR: doxorubicin; *Vp*LAE: *Vernonanthura polyanthes* leaf aqueous extract; *n-*BF: *n-*butanol fraction of *V. polyanthes* leaf aqueous extract.

**Table 2:** Secondary metabolites putatively identified in *Vernonanthura polyanthes* leaf aqueous extract (*Vp*LAE) and its n-butanol fraction (*n*-BF).

Metabolites			*Vp*LAE	*n*-BF
***O*** **-caffeoylquinic acids**				
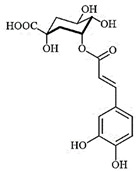	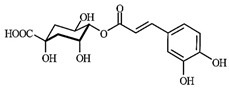	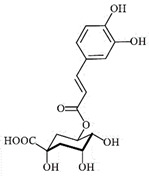		**X**
**3-CQA**	**4-CQA**	**5-CQA**		
***O*** **-feruloylquinic acid**				
	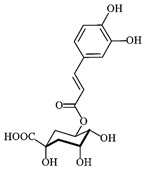		**X**	**X**
	**5-FQA**			
**di-*O*-caffeoylquinic acids**				
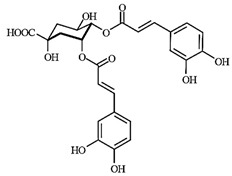	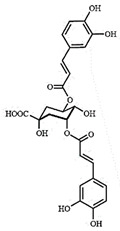	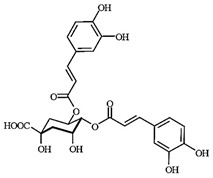	**X**	**X**
**3,4-di-CQA**	**3,5-di-CQA**	**4,5-di-CQA**		
**Flavonoid**				
	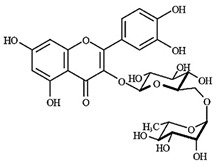		**X**	**X**
	**rutin**			

The X symbol indicates the presence of the metabolite on *Vp*LAE or *n*-BF. 3-CQA, 3-*O-caffeoylquinic* acid; 4-CQA, 4-*O-caffeoylquinic* acid; 5-CQA, *O-caffeoylquinic* acid; 5FQA, 5-*O*-feruloylquinic acid; 3,4-di-CQA, 3,4-di-*O*-caffeoylquinic acid; 3,5-di-CQA, 3,5- di-*O*-caffeoylquinic acid; 4,5-di-CQA, 4,5-di-*O*-caffeoylquinic acid.

**Table 3:** Biological activity prediction of putatively identified metabolites in *Vernonanthura polyanthes* leaf aqueous extract (*Vp*LAE) and its *n-*butanol fraction (*n-*BF) by the PASS online webserver.

	3-CQA		5-CQA		4-CQA		5-FQA		RUTIN		3,4-di-CQA		3,5-di-CQA		4,5-di-CQA	
Biological Activity	*Pa*	*Pi*	*Pa*	*Pi*	*Pa*	*Pi*	*Pa*	*Pi*	*Pa*	*Pi*	*Pa*	*Pi*	*Pa*	*Pi*	*Pa*	*Pi*
Antimutagenic	N/A	N/A	N/A	N/A	0.946	0.001	0.966	0.001	N/A	N/A	0.955	0.001	0.961	0.001	0.955	0.001
Anticarcinogenic	0.846	0.004	0.846	0.004	0.824	0.004	0.863	0.003	0.983	0.001	0.850	0.004	0.837	0.004	0.850	0.004
Antineoplastic	0.778	0.014	0.778	0.014	0.756	0.018	0.792	0.013	0.849	0.007	0.790	0.013	0.777	0.015	0.790	0.013
Chemopreventive	0.833	0.003	0.833	0.003	0.812	0.004	0.875	0.003	0.968	0.001	0.830	0.003	0.827	0.003	0.830	0.003
Antioxidant	0.785	0.004	0.833	0.003	0.771	0.004	0.727	0.004	0.923	0.003	0.806	0.003	0.780	0.004	0.806	0.003
Free radical scavenger	0.856	0.002	0.856	0.002	0.830	0.002	0.913	0.002	0.988	0.001	0.848	0.002	0.841	0.002	0.848	0.002

Pa: probability to be active (Pa > 0.7); Pi: probability to be inactive (Pi > 0.3); 3-CQA: 3-*O*-Caffeoylquinic acid; 4-CQA: 4-*O*-Caffeoylquinic acid; 5-CQA: 5-*O*-Caffeoylquinic acid; 5-FQA: 5-*O*-feruloylquinic; RUTIN: quercetin-3-*O*-rutinoside; 3,4-di-CQA: 3,4-di-*O*-caffeoylquinic acid; 3,5-di-CQA: 3,5-di-*O*-caffeoylquinic acid; 4,5-di-CQA: 4,5-di-*O*-caffeoylquinic acid. N/A: not active.

**Table 4:** Prediction of enzyme modulation by bioactive compounds putatively identified in *Vernonanthura polyanthes* leaf aqueous extract (*Vp*LAE) and its *n-*butanol fraction (*n-*BF).

Enzymatic Activity	3-CQA	5-CQA	4-CQA	5-FQA	RUTIN	3,4-di-CQA	3,5-di-CQA	4,5-di-CQA
CYP1A inducer	N/A	N/A	N/A	N/A	0.980	N/A	N/A	N/A
CYP1A1 inducer	N/A	N/A	N/A	N/A	0.970	N/A	N/A	N/A
CYP3A4 inducer	N/A	N/A	N/A	N/A	0.850	N/A	N/A	N/A
CYP2C9 inducer	N/A	N/A	N/A	N/A	0.830	N/A	N/A	N/A
CYP3A inducer	N/A	N/A	N/A	N/A	0.820	N/A	N/A	N/A
CYP2H substrate	N/A	N/A	N/A	N/A	0.820	N/A	N/A	N/A
UDP-glucuronosyltransferase substrate	0.710	0.710	0.820	0.850	0.970	0.830	0.840	0.830
Aryl sulfotransferase inhibitor	N/A	N/A	0.940	0.997	N/A	0.950	0.950	0.950
Glutathione-disulfide reductase inhibitor	N/A	N/A	N/A	N/A	0.740	N/A	N/A	N/A
Lipid peroxidase inhibitor	0.850	0.850	0.830	0.880	0.999	0.850	0.840	0.850
HMOX1 expression enhancer	N/A	N/A	N/A	N/A	0.750	N/A	N/A	N/A
NADPH oxidase inhibitor	N/A	N/A	N/A	N/A	0.850	N/A	N/A	N/A
β-glucuronidase inhibitor	N/A	N/A	N/A	N/A	0.760	N/A	N/A	N/A
α-glucosidase inhibitor	N/A	N/A	N/A	N/A	0.860	N/A	N/A	N/A

3-CQA:3-*O*-Caffeoilquinic acid; 4-CQA: 4-*O*-Caffeoilquinic acid; 5-CQA: 5-*O*-Caffeoilquinic acid; 5-FQA: 5-*O*-feruloylquinic; RUTIN: quercetin 3-*O*-rutinoside; 3,4-di-CQA: 3,4-di-*O*-caffeoylquinic acid; 3,5-di-CQA: 3,5-di-*O*-caffeoylquinic acid; 4,5-di-CQA: 4,5-di-*O*-caffeoylquinic acid; CYP: cytochrome P450. N/A: not active.

## Supplementary Materials

The following are available online at https://www.mdpi.com/article/10.3390/molecules27082553/s1, Table S1: Spectroscopic data of the putatively identified metabolites in *V. polyanthes* leaf aqueous extract (*Vp*LAE) and its *n*-butanol fraction (*n*-BF), Figure S1: Letter of the Animal Research Ethics Committee of the Federal University of Goiás (CEUA/UFG), protocol number 069/18.
