# Supplementary material for: Phytochemical Composition and Protective Effect of Vernonanthura polyanthes Leaf against In Vivo Doxorubicin-Mediated Toxicity
Source: Molecules. 2022 Apr 15;27(8):2553. doi: 10.3390/molecules27082553 (PMC9032716; doi:10.3390/molecules27082553)
Supplement: Supplementary file 1 [file molecules-27-02553-s001.zip › molecules-1643061-supplementary.pdf]

Supplementary Material

Phytochemical Composition and Protective Effect of *Vernonanthura polyanthes* leaf against in vivo doxorubicin-mediated toxicity

Jamira Dias Rocha [1], Marilia Elias Gallon [2], Abel Vieira de Melo Bisneto [3], Vanessa Cristiane Santana Amaral [1], Luciane Madureira de Almeida [1], Leonardo Luiz Borges [1,4], Lee Chen-Chen [3], Leonardo Gobbo-Neto [2] and Elisa Flávia Luiz Cardoso Bailão [1,*]

[1] Laboratório de Biotecnologia, Campus Central, Universidade Estadual de Goiás, Anápolis, GO, 75132-903, Brazil; jamiradias@gmail.com (J.D.R.); amaral.vcs@gmail.com (V.C.S.A.); luciane.almeida@ueg.br (L.M.d.A.); leonardo.borges@ueg.br (L.L.B.)

[2] Núcleo de Pesquisa em Produtos Naturais e Sintéticos de Ribeirão Preto, Universidade de São Paulo, Av. do Café s/n, Ribeirão Preto, SP, 14040-903, Brazil; mariliagallon@hotmail.com.br (M.E.G.); gobbo@fcfrp.usp.br (L.G.-N.)

[3] Laboratório de Radiobiologia e Mutagênese, Departamento de Genética, Instituto de Ciências Biológicas I, Universidade Federal de Goiás, Goiânia, GO, 74045-155, Brazil; abelmelo8@gmail.com (A.V.d.M.B.); chenleego@yahoo.com.br (L.C.-C.)

[4] Escola de Ciências Médicas e da Vida, Pontifícia Universidade Católica de Goiás, Goiânia GO, 74605-010, Brazil

* Correspondence: elisa.flavia@ueg.br Tel.: +55 (62) 3328-1151

Table S1. Spectroscopic data of the putatively identified metabolites in *V. polyanthes* leaf aqueous extract (*Vp*LAE) and its *n*-butanol fraction (*n*-BF).

ID	Rt	Usual name (CAS number)	MF (Monoisot. Mass)	Positive Ionization Mode		Negative Ionization Mode		UV_{max}
				TIC (*m/z*)	AIF	TIC (*m/z*)	AIF	
**1**	4.9	3-*O*-caffeoylquinic acid (327-97-9)	$C_{16}H_{18}O_9$ (354.0951)	$[M+H]^+$ 355.1026 bp; $[(M+H) - QA]^+$ 163.0392	163.04	$[M-H]^-$ 353.0891 bp; $[2M-H]^-$ 707.1855	353.09; 191.06 bp; 179.03; 135.04	218; 244 sh; 296 sh; 324
**2**	6.0	5-*O*-caffeoylquinic acid (906-33-2)	$C_{16}H_{18}O_9$ (354.0951)	$[M+H]^+$ 355.1027 bp; $[(M+H) - QA]^+$ 163.0391	163.04	$[M-H]^-$ 353.0891 bp; $[2M-H]^-$ 707.1863; $[(M-H) - CAF]^-$ 191.0559	353.09; 191.06 bp; 135.04	218; 244 sh; 295 sh; 326
**3**	6.2	4-*O*-caffeoylquinic acid (87099-73-8)	$C_{16}H_{18}O_9$ (354.0951)	$[M+H]^+$ 355.1027 bp; $[(M+H) - QA]^+$ 163.0392	163.04	$[M-H]^-$ 353.0880	191.06; 179.03; 173.04 bp; 135.04	219; 246 sh; 298 sh; 325
**4**	7.6	5-*O*-feruloylquinic acid (62929-69-5)	$C_{17}H_{20}O_9$ (368.1107)	$[M+H]^+$ 369.1178	177.05; 163.04 bp	$[M-H]^-$ 367.1046	191.05	297; 326
**5**	8.6	quercetin 3-*O*-rutinoside (153-18-4)	$C_{27}H_{30}O_{16}$ (610.1534)	$[M+H]^+$ 611.1609 bp; $[(M+H) - rhamnosil]^+$ 465.1030; $[(M+H) - rhamnosil-glicose]^+$ 303.0498	303.05	$[M-H]^-$ 609.1466 bp	609.15 bp; 301.03	256; 266 sh; 355
**6**	9.4	3,4-di-*O*-caffeoylquinic acid	$C_{25}H_{24}O_{12}$ (516.1268)	$[M+H]^+$ 517.1353 bp; $[(M+H) - H_2O]^+$	163.04	$[M-H]^-$ 515.1213	515.12; 353.09; 191.06; 179.03;	219; 243 sh; 297 sh; 321

ID	Rt	Usual name (CAS number)	MF (Monoisot. Mass)	Positive Ionization Mode		Negative Ionization Mode		UV_{max}
				TIC (*m/z*)	AIF	TIC (*m/z*)	AIF	
		(89886-30-6)		499.1244; $[(M+H) – CAF]^+$ 355.1027; $[(M+H) – QA-CAF]^+$ 163.0387			173.04 bp	
**7**	9.7	3,5-di-*O*-caffeoylquinic acid (89919-62-0)	$C_{25}H_{24}O_{12}$ (516.1268)	$[M+H]^+$ 517.1382 bp. $[(M+H) – H_2O]^+$ 499.1227; $[(M+H) – QA-CAF]^+$ 163.0394	163.04	$[M-H]^-$ 515.1216	353.09; 191.06 bp; 179.03	221; 244 sh; 296 sh; 325
**8**	10.2	4,5-di-*O*-caffeoylquinic acid (89886-31-7)	$C_{25}H_{24}O_{12}$ (516.1268)	$[M+H]^+$ 517.1346 bp; $[(M+H) – H_2O]^+$ 499.1228; $[(M+H) – CAF]^+$ 355.1027; $[(M+H) -QA]^+$ 163.0392	163.04	$[M-H]^-$ 515.1213	515.12; 353.09; 191.06; 179.03; 173.04 bp	219; 244 sh; 299 sh; 327

ID, peak identification; Rt, retention time in minutes; MF, molecular formular; Monoisot. Mass, monoisotopic mass; TIC, Total Ion Chromatogram; AIF, All Ion Fragmentation; UV_{max}, wavelength of maximum absorption in the ultraviolet spectral region; sh, shoulder; QA, quinic acid; CAF, caffeoyl unit; bp, base peak.

MINISTÉRIO DA EDUCAÇÃO
UNIVERSIDADE FEDERAL DE GOIÁS
PRÓ-REITORIA DE PESQUISA E INOVAÇÃO
COMISSÃO DE ÉTICA NO USO DE ANIMAIS/CEUA

UFG

C E R T I F I C A D O

Certificamos que a proposta intitulada **ANÁLISE DAS FRAÇÕES DO EXTRATO AQUOSO DE FOLHAS DE *Vernonanthura polyanthes* QUE POTENCIALIZAM A GENOTOXICIDADE DA DOXORRUBICINA**, registrada com o protocolo **nº 069/18**, sob a responsabilidade de **JAMIRA DIAS ROCHA** que envolve a produção, manutenção ou utilização de animais pertencentes ao filo Chordata, subfilo Vertebrata (exceto humanos), para fins de pesquisa científica (ou ensino) - encontra-se de acordo com os preceitos da Lei nº 11.794, de 8 de outubro de 2008, do Decreto nº 6.899, de 15 de julho de 2009, e com as normas editadas pelo Conselho Nacional de Controle de Experimentação Animal (CONCEA), e foi aprovada pela COMISSÃO DE ÉTICA NO USO DE ANIMAIS (CEUA) da Universidade Federal de Goiás (UFG), em reunião de **17/09/2018**

- ❑ Finalidade: ( ) Ensino (X) Pesquisa Científica
- ❑ Vigência da autorização: 09/2018 a 03/2020
- ❑ Espécie/linhagem/raça: *Mus musculus*/Swiss Webster
- ❑ Nº de animais autorizados: 140
- ❑ Peso/Idade: 20 a 30 gramas, 7 a 12 semanas de idade
- ❑ Sexo: machos
- ❑ Origem (fornecedor): Biotério Central da Universidade Federal de Goiás

Dra. Liliana Borges de Menezes Leite
Vice-Coordenadora da CEUA/PRPI/UFG

Comissão de Ética no Uso de Animais/CEUA
Pró-Reitoria de Pesquisa e Inovação/PRPI-UFG, Alameda Flamboyant, Qd. K, Edifício K2, 1° andar, Prédio da Agência de Inovação, Parque Tecnológico, sala da CEUA, Campus Samambaia – Goiânia-GO, Fone: (55-62) 3521-1876.
Email: ceua.ufg@gmail.com

Figure S1. Letter of the Animal Research Ethics Committee of the Federal University of Goiás (CEUA/UFG), protocol number 069/18.
